# Multi-functional flexible 2D carbon nanostructured networks

**DOI:** 10.1038/s41467-020-18977-6

**Published:** 2020-10-12

**Authors:** Shichao Zhang, Hui Liu, Jianyong Yu, Bingyun Li, Bin Ding

**Affiliations:** 1 State Key Laboratory for Modification of Chemical Fibers and Polymer Materials, College of Textiles, Donghua University, 201620 Shanghai, China; 2 Innovation Center for Textile Science and Technology, Donghua University, 200051 Shanghai, China; 3 Department of Orthopedics, School of Medicine, West Virginia University, Morgantown, WV 26506 USA

**Keywords:** Energy storage, Electrical and electronic engineering, Nanoscale materials, Two-dimensional materials

## Abstract

Two-dimensional network-structured carbon nanoscale building blocks, going beyond graphene, are of fundamental importance, and creating such structures and developing their applications have broad implications in environment, electronics and energy. Here, we report a facile route, based on electro-spraying/netting, to self-assemble two-dimensional carbon nanostructured networks on a large scale. Manipulation of the dynamic ejection, deformation and assembly of charged droplets by control of Taylor cone instability and micro-electric field, enables the creation of networks with characteristics combining nanoscale diameters of one-dimensional carbon nanotube and lateral infinity of two-dimensional graphene. The macro-sized (meter-level) carbon nanostructured networks show extraordinary nanostructural properties, remarkable flexibility (soft polymeric mechanics having hard inorganic matrix), nanoscale-level conductivity, and outstanding performances in distinctly different areas like filters, separators, absorbents, and wearable electrodes, supercapacitors and cells. This work should make possible the innovative design of high-performance, multi-functional carbon nanomaterials for various applications.

## Abstract (web summary)

Creating two-dimensional carbon nanoscale building blocks is of fundamental importance, but is extremely challenging. Here, the authors report a facile electro-spraying/netting technique for self-assembly of two-dimensional carbon nanostructured networks with superflexibility and multi-functionality.

## Introduction

Low-dimensional carbon nanomaterials have significant technological implications for areas in environment, energy, electronics, and healthcare, due to their remarkable mechanical, electronic, optical, and thermal properties^1–4^. Carbon nanoscale building block regimes mainly contain zero-dimensional (0D) nanodots, one-dimensional (1D) nanotubes, two-dimensional (2D) graphene, and their derivatives^5,6^. All these blocks share a common structural characteristic of nanoscale sizes, and their extraordinary properties/performances only occur at the nanoscale, rather than at macro-size^7–9^. Various technologies, such as in situ growth, template synthesis, self-assembly, etc. have been proposed and utilized to construct bulk carbon nanomaterials from their isolated blocks^10–12^. Unfortunately, the unique nanoscale properties often disappear or are greatly compromised when assembled into bulk materials. Nature provides a simple yet efficient avenue towards nanomaterial assembly: an ordered fibrous network structure allows materials to show unique properties/functions^13–16^. The leaf veins, spider webs, honeycombs, etc. are vivid cases. They all possess fibrous networks but are structurally robust, high-performance, and multi-functional. Despite extensive efforts, however, existing carbon fibrous nanomaterials mainly depend on deposition of 1D carbon nanotubes and electrospun nanofibers, in which, the nanotubes suffer from poor continuity (length <100 μm); for the nanofibers, pseudo-nanoscale >200 nm or μm in diameter is inevitable^17–21^. Besides, they both assemble into random-deposited nonwovens lacking of interior linking, rather than into organized networks. Such intrinsic bottlenecks result in their greatly deteriorated performances. The challenge is, therefore, to create continuous, 2D network-structured, and macro-sized carbon nanomaterials without compromising their nanoscale properties.

**Fig. 1:**
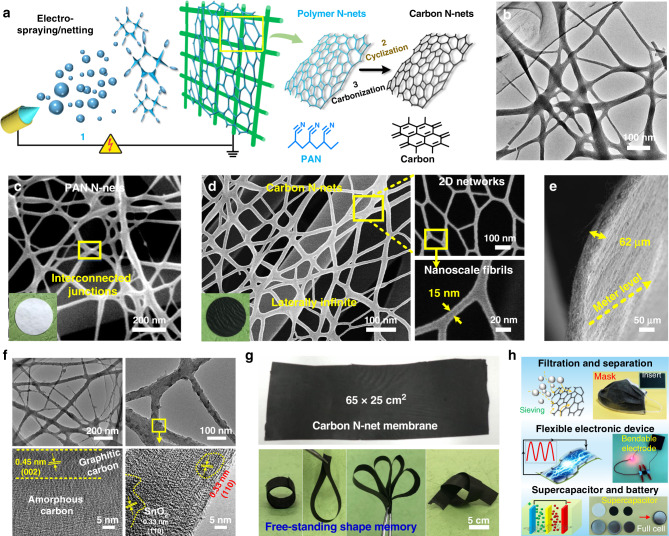
Processing, architectures, and samples of carbon N-nets. **a** Schematic showing the synthetic steps of 2D N-nets. (1) Self-assembly of PAN N-nets using electro-spraying/netting. (2) Cyclization, and (3) carbonization to fabricate carbon N-nets. **b** TEM topography images of the carbon N-nets. SEM images of **c** PAN and **d** carbon N-nets. The N-nets consist of 1D nanoscale fibrils, with structural characteristics of triangular junctions and laterally infinite 2D networks. **e** SEM images showing cross-section view of free-standing carbon N-net membranes. **f** TEM images of the carbon N-nets and their interior nanostructures. **g** A show of large-scale carbon N-net membrane and their flexibility demonstrations. **h** Photographs of carbon N-net samples (mask inserts, bendable electrodes, etc.) and related schematics of their various applications.

Here, we report a unique electrohydrodynamic methodology, electro-spraying/netting, to generate self-assembled 2D carbon nanostructured networks (N-nets) with well interconnected nanoscale fibers (diameters of ~15 nm, Fig. 1). Our approach allows the dynamic ejection and assembly of droplets from Taylor cone to be tailored, making them assemble into ordered network architectures. The carbon N-nets have both characteristics of nanoscale diameters of 1D nanotube and lateral infinity of 2D graphene. Using these unique N-nets as innovative building blocks, we assembled macro-sized (meter level) carbon nanomaterials that presented nanoscale properties, high flexibility (1050 MPa while softer than napkins), conductivity (920 S cm^–1^, as high as single carbon nanofiber), etc. The same nanomaterials exhibited advanced properties for very different applications like filters, separators, absorbents, wearable electrodes, supercapacitors, and cells.

## Results

### Assembly and architectures of carbon N-nets

2D nanostructured networks (N-nets) were self-assembled using our unique electro-spraying/netting technique (Fig. 1a). The formation of N-nets was due to a droplet ejection–deformation–assembly process that underlay Taylor cone instability and differential micro-electric driving. Tailoring of the Taylor cone dynamic and of the micro-electric field enabled the highly charged droplets to eject, self-assemble and deform before they phase separated and evolved into a 2D nanofibrous network (Supplementary Discussion). A facile cyclization and calcination of polymeric (polyacrylonitrile, PAN) precursor N-nets led to the generation of carbon N-nets (Supplementary Fig. 1). A closer transmission electron microscopy (TEM) look (Fig. 1b) revealed that the carbon N-nets had both characteristics of nanoscale diameters similar to 1D carbon nanotube and lateral structures like 2D graphene.

Scanning electron microscopy (SEM) imaging indicated that the PAN N-nets consist of interconnected nanofibers with diameters of 10–40 nm (Supplementary Fig. 2a) and showed 2D topological network structures (Fig. 1c). Besides the unique 2D form, the fiber diameter was true nanoscale, one order of magnitude less than those of conventional nanofibers^22,23^. After oxidization and calcination process, the 2D N-net structures remained (Supplementary Fig. 3) and their fibers decreased to 5–25 nm in diameter (Supplementary Fig. 2b), achieving the diameter level of 1D carbon nanotubes (Fig. 1d). Zooming in and out on the carbon N-nets revealed that a continuous, uniform and integrated network formed and was laterally infinite like but superior to 2D graphene (>meter level compared to <50 μm of graphene in size), as shown in Fig. 1d and Supplementary Fig. 3d. The carbon N-nets are an innovative 2D nanomaterial building block (Fig. 1d, right insets) that is quite different from conventional 1D nanomaterial blocks (nanotube, nanowire, nanorod, etc.) and 2D nanomaterial blocks (graphene, graphitic carbon nitride, transition metal dichalcogenides, etc.)^2,24,25^. Figure 1e displays a free-standing carbon N-net membrane (~62 μm thick) with hierarchical nanostructures (Fig. 1f). Numerous nanoclusters with sizes of 3–12 nm were dispersed in both the carbon matrix and the surface, and caused nanoscale surface roughness (Supplementary Fig. 4). The graphitic carbon layers with inter-planar distance of 0.45 nm was also observed (Supplementary Fig. 5).

Due to the simplicity of our methodology, great versatility in controlling the precursor polymer category and scaling up the assembly of carbon N-nets was feasible. Figure 1g shows a photograph of the carbon N-net membrane with size of 65 × 25 cm^2^; much larger (at meter level) samples could also be produced (Supplementary Fig. 6). Strikingly, the carbon N-net membranes exhibited robust mechanical strength and excellent softness, and could be folded and bent without any damage, behaving as a sheet of rubber (Fig. 1g bottom). Such remarkable properties, for instance, nanoscale characteristics, 2D network form, robust flexibility, and high conductivity, allow the carbon N-nets to facilitate their widespread applications ranging from filtration and separation to wearable electronic device to supercapacitor and battery^26–30^; some industrial samples (Fig. 1h, such as mask and supercapacitor), using carbon N-nets as core components, were also successfully fabricated.

### Synthesis and nanostructures of carbon N-nets

**Fig. 2:**
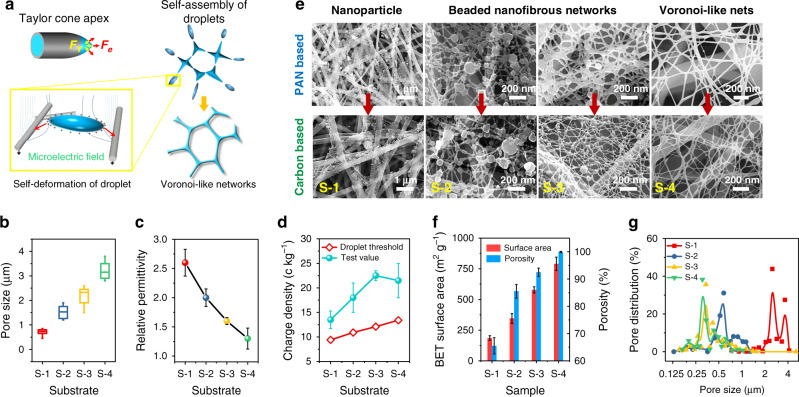
Origin, synthesis, and nanostructural properties of carbon N-nets. **a** Schematic illustration of droplet ejection and its deformation-assembly process during electro-spraying/netting. **b** Pore size and **c** relative permittivity of the utilized substrates, and **d** charge density and droplet threshold of the liquids ejected from precursor solutions and collected using these substrates by electro-spraying/netting. S-1–S-4 indicate the four different substrates or their triggered architectures. **e** SEM images showing the PAN and carbon-based nanoarchitectures formed on different collectors. **f** BET surface area and porosity and **g** pore size distribution of the membranes consisted of different nanoarchitectures. Base weight in **g**, ~0.08 g m^−2^.

To reveal the self-assembly route and control of N-nets, a possible model was proposed (Fig. 2a). Forces leading to droplet ejection from Taylor cone rely on competing Coulomb repulsion and hydrostatic pressure^31,32^. When charge density of the liquid exceeded the droplet threshold *D*_c_, ($\sqrt {288\varepsilon \gamma /\delta \rho ^2D^3}$, see Supplementary Notes 1 and 2), the droplets ejected due to Taylor cone instability. Then the levitating cluster of droplets possibly underwent a self-assembly of spatial pattern driven by the Voronoi dissipative effect^33,34^; meanwhile, they self-deformed driven by the differential micro-electric fields, and resulted in the generation of nanofiber networks with Voronoi-like structures. SEM imaging provided evidence supporting this possible formation process (Fig. 2e). Different collectors triggered the assembly of distinct architectures, including nanoparticles, beaded nanofibers, and Voronoi-like networks.

Using electro-spraying/netting technique, manipulation of the surface topography and conductivity of the collectors (Fig. 2b, c, Supplementary Discussion) enabled the creation of polymeric N-nets. Upon increasing the deposition of PAN fibers (from S-4 to S-1, Supplementary Fig. 7a), which used as collectors, their pore size decreased from 3.2 to 0.7 μm (Fig. 2b), while permittivity increased from 1.25 to 2.6 (Fig. 2c). An increase in pore size of collectors resulted in the distinct transition from nanoparticles to beaded nanofibers to smooth nanofibers due to enhanced potential gradient of micro-electric fields (Supplementary Note 2). The assembly model (Fig. 2d, Supplementary Methods) indicates that, the gap between the charge density and threshold of the droplets was maximized (10.4 c kg^−1^) using S-3 collectors, suggesting the highest probability/speed for droplet formation. Obviously, more nanoarchitectures, i.e., beaded nanofibrous networks, were generated on S-3 collectors (Fig. 2e). In addition, the S-4 collectors induced the creation of Voronoi-like networks because they had both appropriate dielectric property and scaffold pores (Fig. 2a–c).

All carbon-based nanoarchitectures retained their structural characteristics after carbonization from their PAN precursors (Fig. 2e). Compared with the nanoparticles deposited on S-1 substrate (185 m^2^ g^–1^ and 65.5%), both Brunauer–Emmett–Teller (BET)-specific surface area (790 m^2^ g^–1^) and porosity (99.92%) of the carbon N-nets on S-4 substrate were significantly enhanced (Fig. 2f), due to their nanoscale fiber diameters (15 nm, Supplementary Fig. 2) and nanoscale roughness (Fig. 1f and Supplementary Fig. 4). In contrast, most existing electrospun nanofibers typically show BET surface area of <10 m^2^ g^–1^ and porosity of 50–90%. The emergence of 2D N-nets in membranes resulted in extremely small pore sizes of 230–320 nm (Fig. 2g) while maintaining a low thickness of <100 nm; control nanoparticle membrane had pore size of ~3 μm. These structural properties made our carbon N-nets bear great application potential in high-performance filters, sensors, electronics, and optics.

### Properties of carbon N-nets

**Fig. 3:**
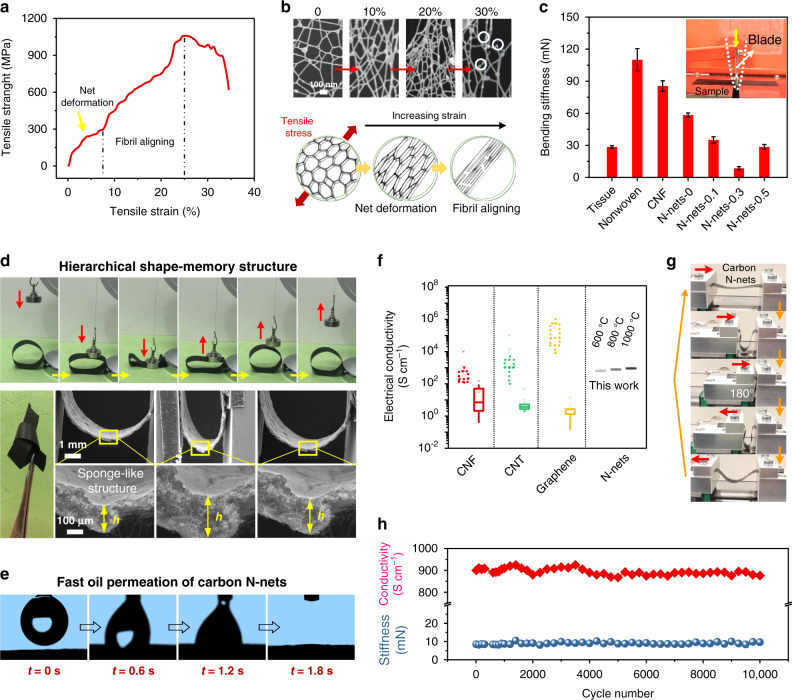
Mechanical properties, surface wettability, and electrical conductivity of the carbon N-nets. **a** Microtensile-strain curve of the free-standing carbon N-nets. **b** SEM images (top) and schematic evolution (bottom) of carbon N-nets during a continuous stretching process. **c** Bending rigidities of the carbon N-nets and selected soft materials. N-nets-X indicates carbon N-nets prepared from PAN solutions with X wt% SnCl_2_. Base weight of N-nets, ~300 mg m^−2^. **d** A set of real-time images showing the flexibility of carbon N-nets (top). SEM observations under cycled compression and release, focusing on a piece of free-standing carbon N-nets (bottom). **e** Optical images of dynamic oil spreading on the surface of carbon N-net membranes. **f** Electrical conductivities of typical carbon nanomaterials (carbon nanofiber or CNF, carbon nanotube or CNT, graphene, and carbon N-nets) in different forms. The solid data boxes indicate assembled bulks, and the dashed data boxes show their isolated building blocks. **g** Photographs showing the cycle fatigue test of the carbon N-nets with bending angle of 180° and **h** their cycling electrical and mechanical performance within 10,000 cycles.

The carbon N-nets were mechanically robust, along with remarkable surface wettability and electrical conductivity. A single-layered, free-standing carbon N-nets exhibited a tensile strength of 1050 MPa with strain of ~25%, indicating two distinct deformation regimes (Fig. 3a). A closer SEM look (Fig. 3b top) at the stretching process of 0–30% *ε*, indicated that the carbon N-nets effectively transferred and dissipated loads. This unique deformation involved net deformation and fibril aligning (Fig. 3b bottom). Tensile stress firstly restricted the nanofibrils in N-nets to align increasingly in a plane along the stretching direction, and led to an undamaged net-shape change, yielding an elastic behavior (0–10% *ε*). Further fibril aligning within 13–20% *ε* allowed for rapidly increased stresses and nonlinear deformation until fragile (>30% *ε*). Moreover, the carbon N-nets showed a bending rigidity of 8.5 mN, which was only 1/10 of polyethylene nonwovens (110 mN) or electrospun carbon nanofiber membranes (85.5 mN). Such performance means that the carbon N-nets possessed soft polymeric mechanical properties while having hard inorganic matrix. The carbon N-nets even had smaller bending rigidity than that (28.5 mN) of tissue paper, suggesting superior softness thereby making it possible to be used as wearable conductive nanomaterials for emerging energy and electronics applications^35^.

The flexibility of the carbon N-nets was further demonstrated by their cycling compression-release evaluation (Fig. 3d). A movie (Supplementary Movie 1) demonstrated that a circular ring made of free-standing carbon N-nets (1.5 × 10 cm^2^) immediately recovered its original shape when lifting a weight of 200 g (300 times heavier than itself), showing striking shape-memory performance^36^. We easily tied a knot using such a carbon N-net membrane. To gain insight into the remarkable flexibility, the hierarchical structures of carbon N-net membranes during deformation were studied by in situ SEM (Fig. 3d bottom). At the top level, the membrane was organized in a lamellar-mesh geometry with sponge-like structure to maximize the stress absorption (thickness *h* increased twice after bending). At the meta-scale level, the 2D interconnected networks, with resilient and restorable deformation capability (20% restorable strain, Fig. 3b), can transfer loading strain with minimum network bending. At the low level, a single carbon nanofiber can bear a large bending deformation without generating any cracks, due to the lubrication matrix structures consisting of amorphous carbon, ordered graphitic carbon and dispersed SnO_2_ nanoclusters (Fig. 1g). Carbon N-nets showed superoleophilicity with an oil contact angle (OCA) of <3°, which was negligible. A high-speed camera visualized their dynamic wetting behavior (Fig. 3e and Supplementary Fig. 8). On the surface, the oil droplet (3 μl, ethylene glycol) spread out quickly (1.8 s) due to the nanocapillary effect^37^. However, the carbon N-nets were superhydrophobic with water contact angles (WCA) of >152°, and they exhibited extremely low water adhesion (Supplementary Fig. 8), revealing self-cleaning capability of wearable electronics^38^.

Despite striking electrical conductivity of isolated nanoscale blocks of carbon materials (such as nanotube and graphene), bringing such remarkable properties to their macroscopic bulks is nearly impossible^39–42^. This challenge is due to the lack of the effective interconnection between isolated blocks. Here, our free-standing carbon N-nets can function as a macroscopic graphene sheet (>meter level). The well graphitized carbon N-nets presented high conductivity (920 S cm^–1^, Fig. 3f), >5 times of those of CNF bulks (1–130 S cm^−1^) and CNT bulks (15–85 S cm^−1^) and similar to those of isolated CNF building blocks (200–1500 S cm^−1^). This was because that the integrated 2D network resulted in a more steadily and continuously conductive path compared to conventional isolated blocks^39,43^. Our carbon N-nets also showed excellent fatigue resistance (Fig. 3g, h and Supplementary Fig. 5). After 10,000 cycle fatigue tests with a completely folded angle of 180° (Fig. 3g and Supplementary Movie 2), the conductivity and stiffness of the carbon N-net membranes (pyrolysis at 1000 °C) remained almost unchanged, maintaining high levels of ~900 S cm^−1^ and 8.8 mN (Fig. 3h), indicating promising application for flexible metacomposites^44–46^.

### Multifunctionality of carbon N-nets

**Fig. 4:**
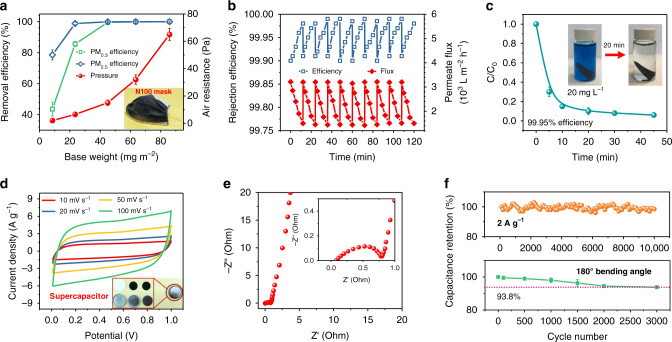
Multifunctionality of combining the air filtration, liquid separation, dye absorption, and electrochemical applications. **a** PM_0.3_ and PM_2.5_ filtration efficiency and air resistance of the carbon N-nets with various base weights. Airflow velocity, 5.33 cm s^−1^. Inset of **a** is the prepared industrial sample of N100 mask using carbon N-nets as core inset. **b** Cycling liquid separation performance of the carbon N-nets driven by gravity. Base weight in **b**, ~320 mg m^−2^. **c** Dye absorption performance of the carbon N-nets. Inset of **c** contains photos showing the dye solutions before and after absorption. **d** CV curves of the carbon N-nets at different scan rates. Inset of **d** is the fabricated supercapacitor using carbon N-nets as electrode. **e** Nyquist plots of the carbon N-nets in the frequency range of 10–10^5^ Hz. **f** Cycling performance of carbon N-nets in supercapacitors at a current density of 2 A g^−1^ (top) and after fatigue test with a bending angle of 180° (bottom).

In considering the small pore size, 2D network structure and high porosity (Figs. 1d, 2f, g), applications in filtration and separation by the carbon N-nets were possible. Particulate matter (PM) pollution in air has raised serious concerns for public health^47–49^. However, most air filters are heavy, bulky, and show inevitable compromise between removal efficiency and air resistance. Our ultrathin carbon N-nets provided an innovative material for high-efficiency and low-resistance air filtration, which was used as filter inset for N100 mask (Fig. 4a inset). Besides PM_2.5_, the carbon N-net filters demonstrated outstanding efficacy for the sternest PM_0.3_ (PM concentration of 300,000–500,000) removal (Fig. 4a). Carbon N-net filters with 45 mg m^−2^ showed efficiency 99.533% for PM_0.3_ removal and 99.925% for PM_2.5_ removal at pressure drop of 15 Pa. A 99.992% efficiency of PM_0.3_ removal and 99.999% efficiency (ultralow penetration air level) of PM_2.5_ removal by N-net filters were achieved at a pressure drop of 65 Pa, exhibiting a higher quality factor (even 0.35 Pa^−1^, Supplementary Fig. 9) and better performance than most existing filters. With increasing PM concentration to 20 million, the PM removal capability of the carbon N-nets was slightly decreased, for instance, from 99.992 to 99.985% for PM_0.3_ removal (Supplementary Fig. 10). The air resistance was only 0.06% of the atmospheric pressure, which was negligible. In addition, their base weights were about one order of magnitude less than those of conventional nanofiber filters (2–30 g m^–2^), and were negligible compared to conventional microfiber filters (usually >100 g m^–2^). We believe that the robust removal efficacy of carbon N-nets rely on small pore size and high interface energy, and their low air resistance is due to high porosity and the slip-effect for airflow from high Knudsen number of 8.8 (Supplementary Table 1).

Due to the numerous channels stemmed from the highly porous architecture and extended surface area (Fig. 2f), water treatment (liquid separation and dye absorption, Fig. 4b, c) by carbon N-nets was expected. Solely by gravity-driven, the carbon N-net membrane (320 mg m^–2^) exhibited rejection efficiency of >99.90% and permeate flux of 3200 l m^–2^ h^–1^ (Supplementary Fig. 11). Such fluxes were >10 times higher than those of microfiltration and ultrafiltration membranes with similar efficiency levels. With increasing separation time, the rejection efficiency increased from 99.90 to 99.99%, and the permeate flux reduced from 3200 to 1520 l m^–2^ h^–1^ (Fig. 4b). This result suggested that the carbon N-nets functioned in a surface filtration manner, leading to the generation of filter cakes. Therefore, after a facile cleaning with water, their separation efficacy was recovered completely (even after 10 cycles, Fig. 4b), a characteristic that has rarely been achieved in present separation membranes but is desirable for cross-flow filtration. The carbon N-nets also could be used as a promising adsorbent for organic pollutant absorption^50^; a small piece of carbon N-net membrane (3 mg) could adsorb most of methylene blue, a pollutant model (20 mg l^–1^, 30 ml), in 20 min, and achieved 99.95% efficiency within 45 min (Fig. 4c). Such a high adsorption of 200 mg g^–1^ could be attributed to their hierarchical roughness (Fig. 1f) and enhanced surface area (Fig. 2f).

The carbon N-nets offered robust conductivity for energy storage and transfer (Fig. 3f), besides, their flexible property also allowed them to be used for wearable electronics (Fig. 3g, h and Supplementary Movie 2). The direct use of free-standing carbon N-nets as electrodes avoided the requirement of loading binders or conductive additives that restrained fast discharge/charge^51^. Here, we assembled all carbon supercapacitors using carbon N-net symmetric electrodes (Fig. 4d). The obtained supercapacitor showed a typical rectangular cyclic voltammograms (CV) curve with redox peaks. This result indicated that the electrical-double-layer capacitance and pseudocapacitance functioned together, which might be arisen from the carbon matrix and doped SnO_2_, respectively. Increasing scan rate (10–100 mV s^–1^) did not change the CV curve shape, indicating the high rate capability. The carbon N-net supercapacitor showed a specific capacitance of 275 F g^–1^ at the rate of 10 mV s^–1^, achieving remarkable performance like the reported hierarchical activated carbon supercapacitors^52^. Figure 4e and Supplementary Fig. 12 present the electrochemical impedance data. The straight line of Nyquist plot (10–10^5^ Hz) of the carbon N-nets in the low frequency region indicated high ionic accessibility, with an equivalent series resistance of 0.08 Ω, suggesting a low internal resistance of the whole cell. In contrast to carbon nanofibers (29 Ω, Supplementary Figs. 7b and 12), the charge resistance transfer R_CT_ of carbon N-nets was only 0.78 Ω, demonstrating striking transmission of electrons and ions. The galvanostatic charge/discharge (0.5–4 A g^–1^, Supplementary Fig. 13) of carbon N-net supercapacitors also showed a symmetrical triangular shape with slight nonlinearities and a short charge-discharge time, confirming its high reversibility. The cycling performance at 2 A g^–1^ (Fig. 4f top) indicated that the N-net supercapacitors retained >96% of the initial capacitance after 10,000 cycles. More strikingly, the used carbon N-net electrodes maintained 93.8% of their original capacitance even after being bent to the angle >180° 3000 times (Fig. 4f bottom), revealing their intriguing application potential for wearable electronics.

## Discussion

The synthesis of carbon N-nets provides an innovative 2D building block, which is quite different from nanomaterial 1D blocks (nanotube, nanowire, etc.) and 2D blocks (graphene, transition metal dichalcogenides, etc.), to develop high-performance carbon nanomaterials. The carbon N-nets showed ordered nanofibrous networks and were laterally infinite, which enabled them to be used directly in pseudo-3D macroscopic form or as core fillers for high-performance nanocomposites^53,54^. Considering the ease of scalable synthesis of 2D N-nets using electro-spraying/netting, our findings pave the way for new types of carbon-based N-nets from various polymeric precursors (such as cellulose, poly(amic acid), polyimide, etc.) for use in diverse applications. Moreover, similar to the carbonization, a variety of oxide ceramic N-nets could be processed by combining with a sol-gel process, and would find widespread use in energy and electronics. In addition, various functions (e.g., photoactive, antimicrobial, catalytic, and magnetic properties), desirable for applications like catalysis, tissue engineering, drug delivery, etc., could be easily endowed with composite N-net materials simply by incorporating functional fillers in precursor solutions or by post-coating on the carbon N-net scaffolds.

In concert, our studies present an innovative self-assembly strategy for the scalable synthesis of carbon N-nets with 2D network structures by unique electro-spraying/netting technique. Such pure nanoarchitectured fibrous networks show the integrated properties of nanoscale diameters (5–25 nm) like 1D carbon nanotube and lateral infinity like 2D graphene. Our data suggest that these carbon N-nets demonstrate various remarkable properties, such as nanostructural characteristics, robust mechanical flexibility, unique surface wettability and superior electric conductivity. As expected, the carbon N-nets yielded excellent performances towards multi-functional applications of air filtration, liquid separation, dye absorption, and electrochemical applications. This work paves the way for creating such exceptional carbon nanomaterials and opens up numerous opportunities for applications in environment, energy, electronics, catalysis, and tissue engineering.

## Methods

### Fabrication of carbon N-nets membranes

The electro-spraying/netting process was performed using a conventional DXSN spinning machine (SOF Nanotechnology Co., Ltd., China). The detailed fabrication process and solution properties are presented in Supplementary Methods and Supplementary Table 2. The used precursor solutions were prepared by dissolving 3 wt% PAN (*M*_w_ = 50,000) in *N,N*-dimethyformamide/stannous chloride (DMF/SnCl_2_) solvents. The SnCl_2_ concentrations in solutions were tuned to 0, 0.1, 0.3, and 0.5 wt%. The solutions were ejected with feed rate of 0.1 ml h^−1^ to form levitating clusters of charged droplets powered by a DC voltage of 30 kV with distance of 15 cm. Designed substrates, nanofiber membranes electrospun from 13 wt% PAN solutions with different spinning durations, were used as collectors to collect the resultant architectures. After vacuum drying (50 °C for 1 h), the as-prepared PAN N-nets were stabilized by oxidization in air at 280 °C for 2 h. Then carbonization process protected in 99.999% N_2_ flow for 2 h was utilized to fabricate carbon N-nets. The temperatures were 600, 800 and 1000 °C with a heating rate of 2 °C min^−1^.

### Characterization

Solution properties including viscosity, conductivity and surface tension, and the charge density of the droplets ejected from metal sprayers during electro-spraying/netting were examined as depicted in Supplementary Methods. The morphologies and structures of the nanostructured architectures were characterized using SEM (Hitachi S-4800, precoated with gold for 150 s) and TEM (JEM-2100F). The thickness and base weight of the carbon N-nets were measured using Labthink thickness gauge (CHYC2, readability of 0.1 μm) and Mettler Toledo Micro balance (AT-20, readability of 2 μg), respectively. The specific surface area and pore structure of the carbon N-nets were characterized by BET ASAP2020 surface area analyzer (Micromeritics Co., USA) and CFP-1100AI capillary flow porometer (Porous Materials Inc., USA). The surface wettability of the carbon N-nets was examined using a contact angle goniometer (SL200B), and their mechanical property data were collected using single-nanofiber mechanical tester (FSF001.1, precision of 1 μN), programmable sliding table and softness tester (RRY-1000). The electrical conductivity of the carbon N-nets was determined with multi-functional four-point probe apparatus (ST-2258C). The filtration performance, such as removal efficiency and air resistance of the carbon N-net-based masks was measured using LZC-G filter equipment (Huada Filter Technology Co., Ltd., China), the testing details are shown in Supplementary Methods. The liquid separation and dye absorption tests of the carbon N-nets were carried out using 100 ppm TiO_2_ nanoparticle suspension and 20 mg l^–1^ MB solution, respectively. The dye and nanoparticle concentrations in solutions were evaluated using UV-vis spectrometer (PG 2000+) with an integrating sphere. The electrochemical measurements were performed in a two-electrode system using electrochemical workstation (Biologic VMP3). CR2025-type coin cells, which used carbon N-net membranes as self-supported electrodes, were utilized and detailed in Supplementary Methods.

## Supplementary information

Supplementary Information

Supplementary Movie 1

Supplementary Movie 2

## Data Availability

The experimental data that support the findings of this study are available from the corresponding author upon reasonable request.
