# Supplementary material for: Multi-functional flexible 2D carbon nanostructured networks
Source: Nat Commun. 2020 Oct 12;11:5134. doi: 10.1038/s41467-020-18977-6 (PMC7550567; doi:10.1038/s41467-020-18977-6)
Supplement: Supplementary file 1 — Supplementary Information [file 41467_2020_18977_MOESM1_ESM.pdf]

Multi-Functional Flexible 2D Carbon Nanostructured Networks

Zhang *et al.*

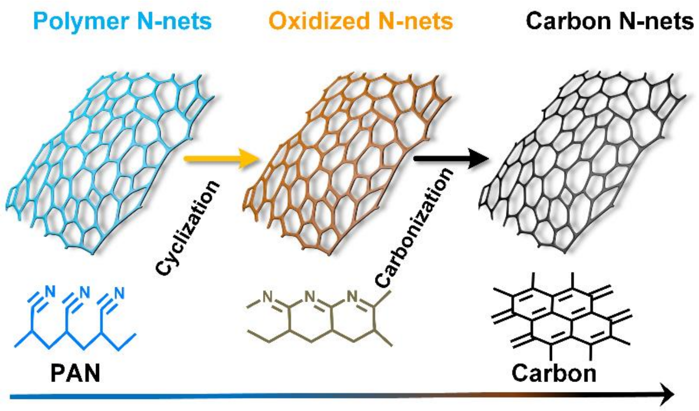

Supplementary Fig. 1 Schematic showing the synthetic steps of 2D carbon N-nets from their precursor polymeric N-nets.

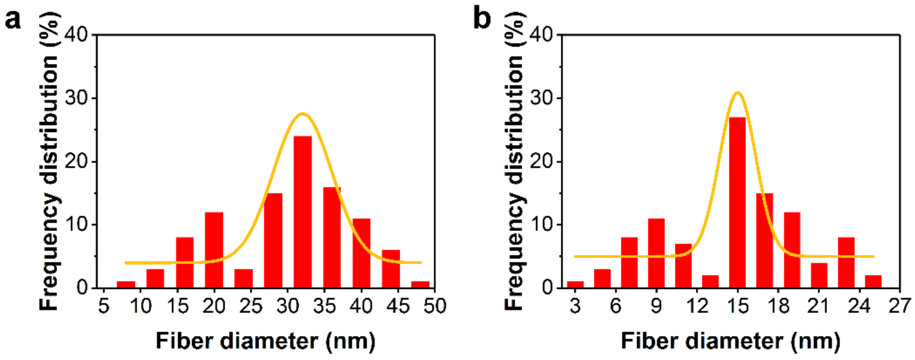

Supplementary Fig. 2 Histogram showing the fiber diameter distribution of (a) PAN N-nets and (b) carbon N-nets.

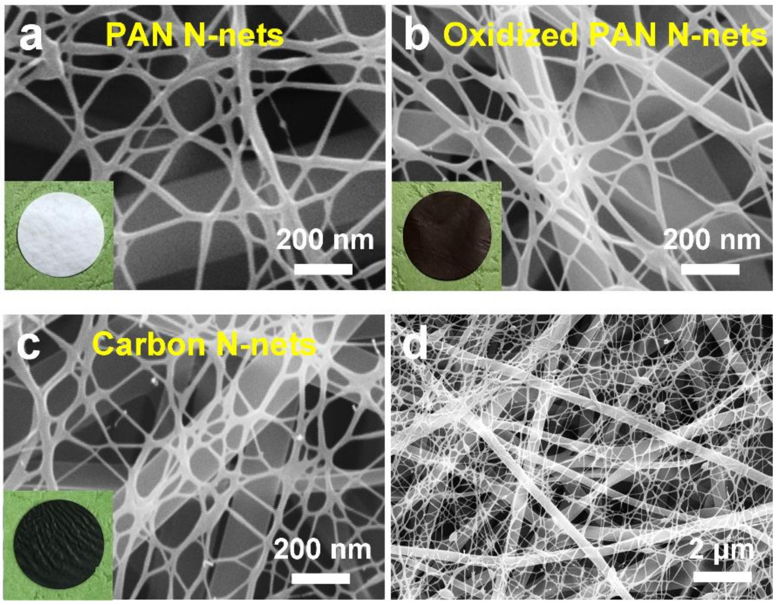

Supplementary Fig. 3 High-magnification SEM images of (a) PAN, (b) oxidized PAN and (c) carbon N-net membranes. Low-magnification SEM showing 2D network structures of carbon N-nets.

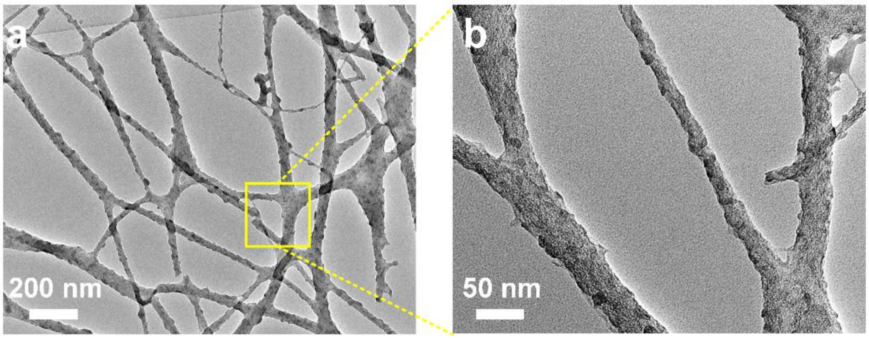

Supplementary Fig. 4 TEM images showing (a) carbon N-nets and (b) their nanoscale surface roughness.

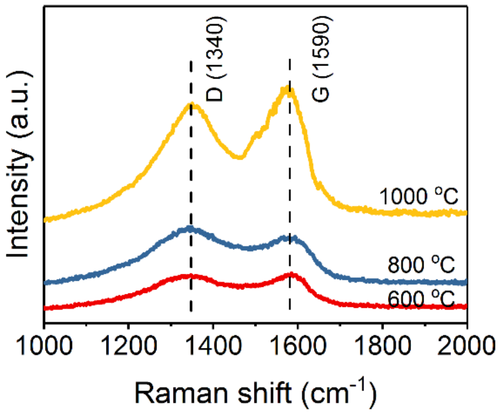

Supplementary Fig. 5 Raman spectra of the carbon N-nets derived from various carbonization temperatures. All the carbon N-nets exhibited typical characteristic bands of 1340 cm^{-1} (D band) and 1590 cm^{-1} (G band). The D band is related to the disordered carbonaceous matrix, resulting from the vibrations of carbon atoms with sp3 bonds in the crystal lattice or from defects in the curved graphene sheets. The G band is associated with the in-plane stretching of sp2 bonded carbon atoms with an ordered graphitized structure. Therefore, the graphitization of the carbon N-nets was enhanced with the increase of carbonization temperatures.

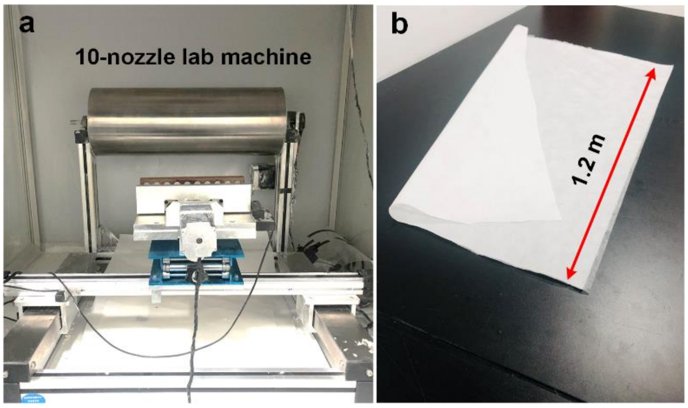

Supplementary Fig. 6 Photographs of (a) the lab machine and (b) the large-sized nanofiber membranes.

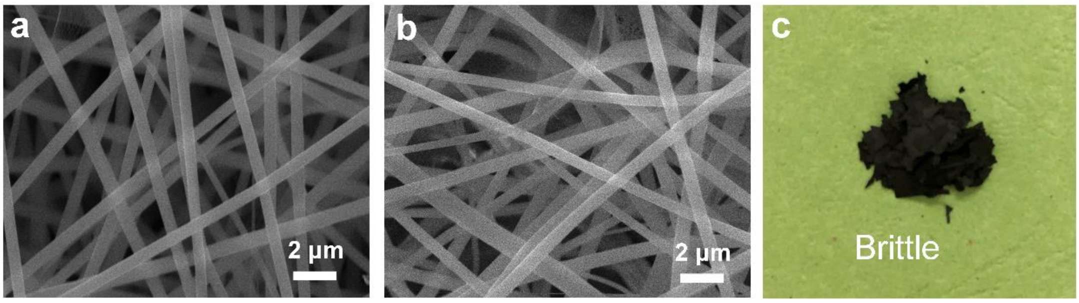

Supplementary Fig. 7 SEM images of (a) PAN substrate nanofibers and (b) their formed carbon nanofibers. (c) The photograph showing the brittle carbon nanofiber membrane.

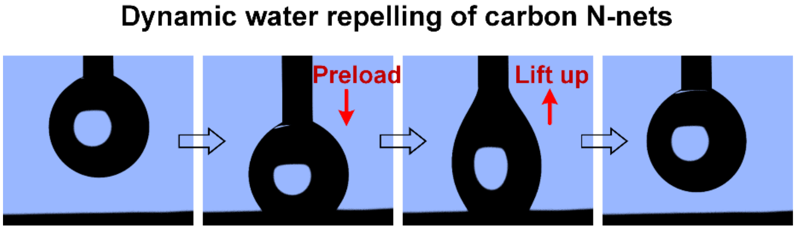

Supplementary Fig. 8 Photographs of dynamic measurement of water adhesion on the carbon N-nets.

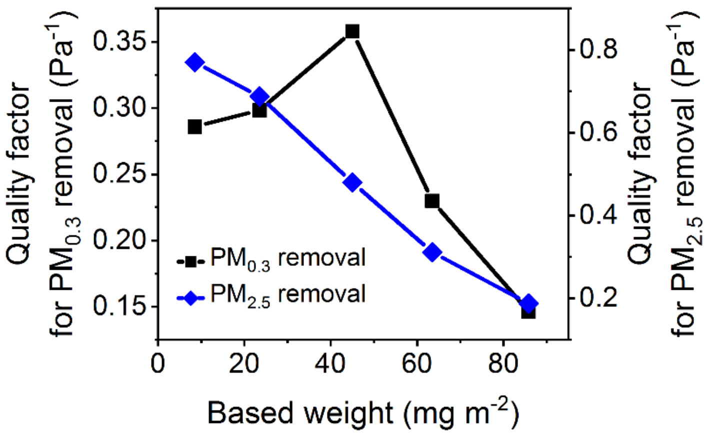

Supplementary Fig. 9 Quality factor of the carbon N-net air filters with various base weights for NaCl $PM_{0.3}$ and $PM_{2.5}$ capture. Airflow velocity, 5.33 cm s^{-1}. The quality factor (*QF*), as a trade-off indicator to evaluate the filtration capacity of air filters, is defined by the formula: $QF = -\ln(1 - \eta)/\Delta p$, in which η is removal efficiency and Δp is pressure drop.

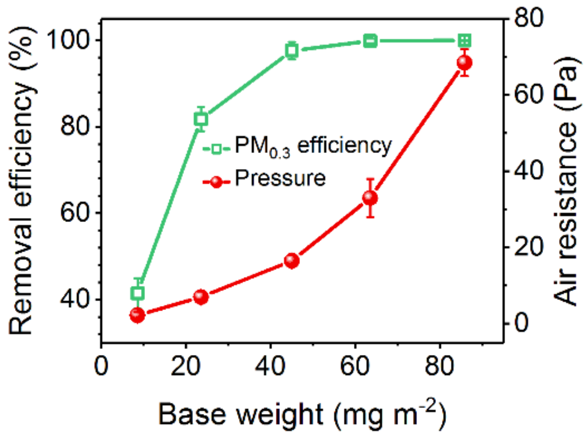

Supplementary Fig. 10 $PM_{0.3}$ removal efficiencies and pressure drops of the carbon N-net filters with various base weights (PM number concentration of 20 million). With increasing PM concentration from 0.3–0.5 to 20 million, the carbon N-nets exhibited a slightly decreased efficiency for $PM_{0.3}$ removal, for example, from 99.935% to 99.891% for 63.5 mg m^{-2} filters and from 99.992% to 99.985% for 85.8 mg m^{-2} filters (Fig. 4a). This steady removal capacity was attributed to the unique sieving manner and airflow 'slip-effect' originating from nanostructured networks.

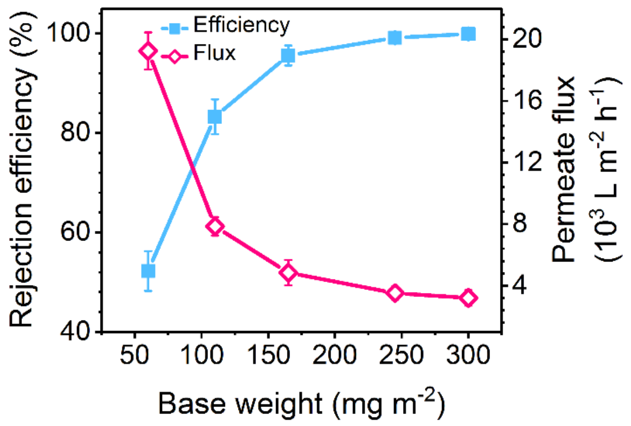

Supplementary Fig. 11 Rejection efficiencies and permeate fluxes of the carbon N-net membranes with various base weights. By using a dead-end filtration device, 100 ppm TiO_2 nanoparticle (diameter 200–400 nm) suspension was poured on the prewetted carbon N-net membrane to test the separation performance. With increasing the base weight (50–320 mg m^{-2}), the carbon N-nets showed a sharply increased removal efficiency from 55% to 99.9% together with a decreased permeate flux from 20,000 to 3200 L m^{-2} h^{-1}.

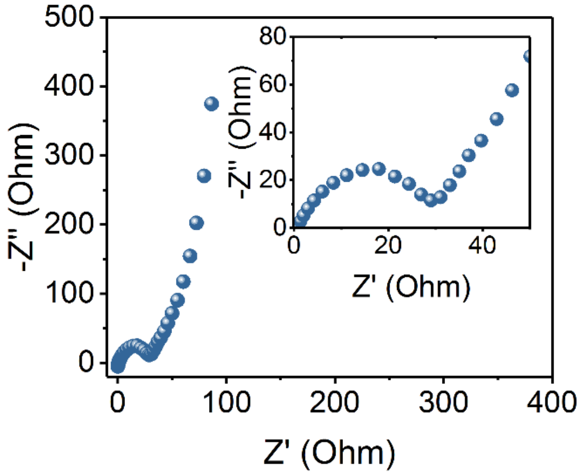

Supplementary Fig. 12 Nyquist plots of the control carbon nanofiber membranes in the frequency range of 10 to 10^5 Hz.

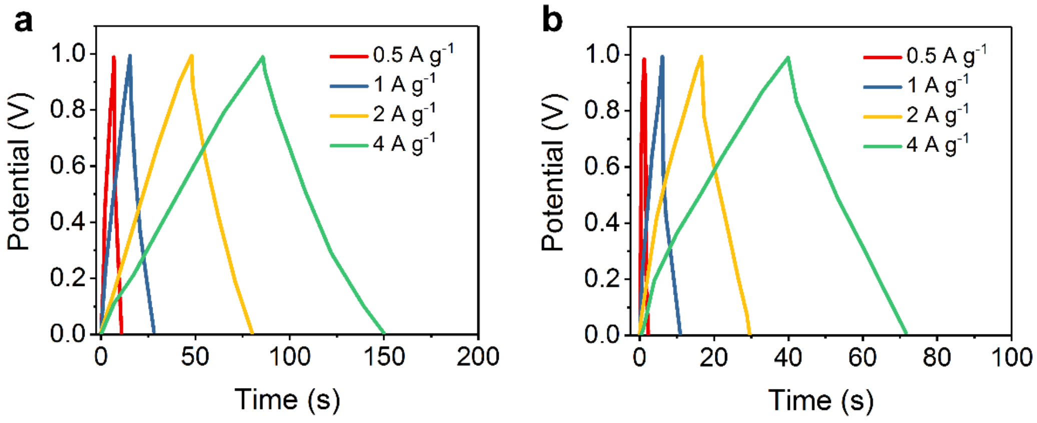

Supplementary Fig. 13 Charge–discharge curves of (a) the carbon N-nets and (b) the control carbon nanofibers at various current densities.

Supplementary Table 1 Knudsen numbers and flow regimes dependent on different ranges of fiber diameter under normal conditions.

**Knudsen number (Kn)**	**Fiber diameter (*d*)**	**Flow regimes**
Kn < 0.001	d > 132 μm	Continuum flow regime
0.001 < Kn < 0.25	528 nm < d < 132 μm	Slip flow regime
0.25 < Kn < 10	13.2 nm < d < 528 nm	Transition flow regime
Kn > 10	d < 13.2 nm	Free molecular flow regime

The Knudsen number (Kn) is a parameter used to define the flow regime of air flow, and can be calculated by Kn = $2\lambda/d$, in which, λ is the mean free path of air molecules (66 nm under normal conditions). Generally, when the diameter of the fiber is comparable to λ, the airflow velocity is non-zero at the fiber surface because "slip" occurs. Thus the air resistance is greatly reduced. The slip-effect for airflow starts to work at Kn ≥0.001 and is sharply enhanced when Kn ≥0.25. The carbon N-nets have average diameter of 15 nm, thus show a high Kn of ~8.8. This result indicates that the carbon N-nets can operate in the transition flow regime, even approach the free molecular flow regime, resulting in the low air resistance.

Supplementary Table 2 Compositions and properties of PAN precursor solutions.

**PAN (wt%)**	**$SnCl_2$ (wt%)**	**Viscosity (cps)**	**Conductivity (mS/cm)**	**Surface tension (mN/m)**
3	0	8.01	0.15	35.5
3	0.1	8.23	1.26	35.8
3	0.3	8.52	2.55	36.1
3	0.5	8.65	4.52	36.3

Supplementary Discussion

We attributed the electro-spraying/netting process mainly to the extremely diluted precursor solutions of high-molecular-weight polymers and the designed substrates composed of PAN nanofiber membranes. The use of diluted precursor solutions was aimed to achieve the electro-spraying process rather than conventional electrospinning process. While the nanofiber membrane substrates were intended to enable the generation of micro-electric fields to facilitate electro-netting process. The contributions of the nanofiber membrane substrates mainly include two aspects: (i) Different from conductive collectors, the dielectric PAN nanofiber membranes enhanced the charge density of the precursor solutions and resulted in the Taylor cone instability, to generate the charged droplets for subsequent network assembly. (ii) The surface topography (porous structures with empty spaces) of nanofiber membrane substrates enabled the potential gradient of the micro-electric fields, which can facilitate the deformation of droplets (Fig. 2a). Taken together, after the droplet ejection, the levitating cluster of droplets possibly underwent a self-assembly of spatial pattern driven by Voronoi dissipative effect; meanwhile, they self-deformed driven by the differential micro-electric fields, finally resulting in the generation of network structures.

Supplementary Note 1

**Nomenclature**			
e	charge of fluid	x	distance between studied location and center of charged fluid
m	mass of fluid	F_γ	hydrostatic pressure of fluid
e/m	charge density of fluid	F_e	Coulomb repulsion of fluid
ε	ambient permittivity	E	electric field intensity
γ	surface tension of fluid	V	electric potential of fluid
ρ	density of fluid	W	electric energy of fluid
D	diameter of charged fluid/Taylor cone apex	dW	change in electric energy of fluid
R	radius of charged fluid/Taylor cone apex	Q	volumetric flow rate
δ	correction factor	η	viscosity of fluid
K	conductivity of fluid	D_c	droplet threshold

Supplementary Note 2

Ejection of Taylor cone. Here, we chose the diameter of the Taylor cone apex at the maximum curvature as the characteristic diameter of the ejected fluid. This diameter can be calculated by the following parametric equation.

$$D = 1.46Q^{0.44}\varepsilon^{0.12}\eta^{0.32}K^{-0.12}\gamma^{-0.32} \quad (1)$$

Considering the tiny weight of the ejected liquid, the effect of gravity is negligible. Therefore, the ejection are mainly driven by the competition between Coulombic repulsion F_e and hydrostatic pressure F_γ . When $F_e > F_\gamma$, the ejection on Taylor cone occurs. Different from conventional electrospraying process which usually uses high-concentration solutions of low-molecular-weight

polymers, highly charged droplet is assumed to eject from the Taylor cone of high-molecular-weight, low-concentration polymer solution. Thus, the F_γ of the droplet can be calculated by:

$$F_\gamma = {}^{2\gamma}/_{R} \tag{2}$$

The electric field intensity E can be deduced based on Gauss's law:

$$E = {}^{\delta e}/_{4\pi\varepsilon x^2} \tag{3}$$

And the electric energy (W) at the surface of the droplet and its change (dW) when the radius is increased from R to R + dR can be obtained:

$$W = \int_0^e \int_R^\infty E dx\, de = \frac{-e^2}{8\pi\varepsilon R^2} dR \tag{4}$$

$$dW = \frac{-\delta e^2}{8\pi\varepsilon R^2} dR \tag{5}$$

According to the work-energy theorem, the dW can also be calculated:

$$dW = -4\pi R^2 F_e dR \tag{6}$$

Then,

$$F_e = {}^{\delta e^2}/_{32\varepsilon\pi^2 R^4} \tag{7}$$

In addition, the mass of the charged droplet m is:

$$m = \frac{1}{6}\rho\pi D^3 \tag{8}$$

Therefore, the droplet threshold can be deduced by Supplementary Equations (1), (2), (7) and (8):

$$D_c = \sqrt{{}^{288\varepsilon\gamma}/_{\delta\rho^2 D^3}}$$

By analyzing theoretical thresholds and experimental data (Supplementary Methods), the

prediction diagram for the assembly of N-nets was established (Fig. 2d). Limited pore size (S-1) led to the formation of nanoparticles, since there was typically no self-deformation of droplets due to the weak potential gradient of micro-electric fields.

Supplementary Methods

Measurement of the charge density of the ejected liquids

The typical method of "mesh target" which widely used for electrospray technology was introduced to measure the charge density of the droplets during electro-spraying/netting process. A high precision multimeter (Fluke F15B+) was utilized to connect the collector and the ground terminal of high-voltage power supply, and used to test the current induced by flying charged droplets. During a certain time (t), the total charge (e) can be obtained by: $e = I \times t$, where I is the average current value. And, the mass (m) of the droplets in this period can be calculated by: $m = M/c$, in which M indicates the mass of the N-net membrane and c is the PAN/$SnCl_2$ concentration. Here, the current was recorded once per 30 s, and at least 5 parallel tests were carried out for each kind of systems.

Uniformity control of nanofiber membranes

During electro-spraying/netting process, to control membrane uniformity, five syringes were uniformly set on an injection pump which horizontally moved backwards and forwards with speed of 200 cm min^{-1} and within a fixed distance, by using a mechanical slide unit. And, an electric shield device on each needle was employed to ensure that the droplets flew forward, thus the nanomaterials (nanoparticles, nanofibers or fibrous networks) could be uniformly deposited during the quadrature motion process caused by the synchronous movement of the stainless roller and mechanical slide unit.

Filtration measurement

In this work, the LZC-G filter tester (Huada Filter Technology Co., Ltd.) was used to evaluate the filtration performance of the air filters. The aerosol particles with diameters of 0.3−10 μm and a geometric standard deviation of <1.86 were generated from 2 wt% NaCl aqueous solutions and were used as PM objects after they were charge-neutralized. The filter membrane with area of 100 cm^2 were

clamped on the filer hold, 0.3–0.5 or 20 million PM were delivered through the filter by the air pump. Using two laser particle counters, the removal efficiency can be calculated by detecting the number of PM in the upstream and downstream of the particle airflow. Similarly, the pressure drop of the filters can be measured by two electronic pressure transducers. A minimum of five testing regions were measured for each kind of filter membranes (size of 65 × 25 cm^2) to ensure the representativeness and reliability.

Electrochemical measurement

Here, the electrochemical measurements were carried out in two electrode system using an electrochemical workstation (Biologic VMP3). The self-standing carbon N-net membranes were utilized as electrodes to assemble the two-electrode symmetric coin cells. 1M H_2SO_4 solution was used as the aqueous electrolyte. Cyclic voltammetry (CV) tests were carried out at different scan rates ranging from 10 to 100 mV s^{-1}, while the constant current charge–discharge measurements were conducted by chronopotentiometry (CP) at 0.5 to 4 A g^{-1}. All the measurements were carried out at room temperature.
